# Numerical and Analytical Study of Two-Layered Unsteady Blood Flow through Catheterized Artery

**DOI:** 10.1371/journal.pone.0161377

**Published:** 2016-08-22

**Authors:** Akbar Zaman, Nasir Ali, M. Sajid, Tasawar Hayat

**Affiliations:** 1 Department of Mathematics and Statistics, Faculty of Basic and Applied Sciences, International Islamic University, Islamabad, 44000, Pakistan; 2 Department of Mathematics, Quaid-I-Azam University, Islamabad, 4400, Pakistan; 3 Nonlinear Analysis and applied Mathematics (NAAM) Research Group, Department of Mathematics, Faculty of Science, King Abdulaziz University, Jeddah, 21589, Saudi Arabia; Bioinformatics Institute, SINGAPORE

## Abstract

The pulsatile flow of blood in a catheterized blood vessel is analyzed. The flow of blood in vessel is modeled as the flow of two immiscible fluids. The fluid in the core region is characterized as a non-Newtonian viscoelastic fluid satisfying the constitutive equation of an Oldroyd-B fluid. The fluid in the peripheral region is treated as a Newtonian fluid. The catheter inside the vessel is modeled as a rigid tube of very small radius. The resulting differential system for velocity in each region is computed numerically by finite-difference scheme and analytically by Laplace transform. A comparison of numerical solution with Laplace transform solution is carried out. Various physical quantities of interest through the computed velocity are also analyzed.

## 1. Introduction

Medical catheters are used in medical centers to insert fluids or gases to patients or to deplete bodily fluids such as urine. Apart from that they are utilized for the diagnosis and treatment of various arterial diseases. Moreover, the monitoring of velocity and pressure gradient during any treatment/diagnostic procedure is also achieved by using catheters. Despite their advantages, there are certain consequences of clinical significance attached with their usage. It is observed that the insertion of catheter in an artery affects the flow field, disturb the pressure distribution and enhance the resistance to flow. Therefore it is necessary to study such increase in flow variables due to catheterization.

Very few attempts have been made in the past to study the blood flow through a catheterized artery. Back [1] estimated the resistance to the flow by assuming that the blood as a Newtonian behavior. He concluded that even a very small size of angioplasty guide-wire leads to sizable increase in flow resistance. In another study Back and Denton [2] provided the estimates of wall shear stress in coronary angioplasty and discussed its clinical implications. MacDonald [3] investigated the characteristics of blood flow in catheterized arteries using finite difference technique. Some possible effects of catheter on arterial wall are studied by Karahalios [4]. He observed that such effects become more significant when the annular gap between catheter and artery becomes narrow. The influence of catheterization on blood flow in a curved artery was discussed by Jayaraman and Tiwari [5].

The simultaneous effects of non-Newtonian nature of blood and catheter on flow characteristics are also reported by some researchers. For instance Dash et al. [6] utilized constitutive equation of Casson model to estimate the increase in flow resistance due to catheter in a narrow artery for both steady and pulsatile flow cases. The analysis of Dash et al. [6] was extended by Sankar and Hemalatha [7, 8] for Herschel-Bulkley model.

On the other hand the unsteady blood flow through stenosed arteries is also attended by several investigators. For instance, Mekheimer and El Kot [9] analyzed the effects of Hall current on blood flow through an artery under stenotic conditions. Mustapha et al. [10] examined the characteristics of arterial constrictions and body acceleration in unsteady blood flow. In another attempt, Mustapha et al. [11] explored the blood flow in irregular multi-stenosed arteries. Here emphasis is given to the effect of applied magnetic field. A mathematical model for generalized Newtonian blood flow with irregular arterial stenosis is also developed and simulated by Mustapha et al. [12]. Abdullah et al. [13] addressed the influence of magnetic field on blood flow with irregular stenosis. A mathematical model for axisymmetric artery via cosine shaped stenosis is computed by Shahed et al. [14]. Apart from above mentioned studies, some investigators have also explored the effects of catheter on Newtonain and Non-Newtonian flows through stenosed arteries. Mentioned may be made to the studies carried out by Sarkar and Jayaraman [15], Dash et al. [16], Reddy et al. [17], Srivastava and Rastogi [18] and Muthu et al. [19].

It is noted from the available literature that blood flowing through catheterized arteries is taken as a single-phase Newtonian or non-Newtonian fluid. However, due to accumulation of red blood cells in the center of the larger arteries, a cell-depleted layer exists near the vessel wall. To account for such a situation, many researchers have treated the blood as a two-phase fluid where the core region is modeled as non-Newtonian fluids while the cell-depleted layer is assumed as a Newtonian liquid. Additionally there are experimental evidences that blood exhibits viscoelastic properties under certain conditions [20, 21]. Thurston [22] was the first to identify the viscoelastic nature of blood. He developed an extended Maxwell model, which is applicable to one-dimensional flow [23]. Yeleswarapu et al. [24] and Yeleswarapu [25] proposed an Oldroyd-B fluid model with three parameters for studying the blood flow. It is generally accepted that blood is slightly viscoelastic, and its effect was ignored in most of the computational fluid dynamics studies. At low shear rates, aggregate of RBCs behave like solid bodies and has abilities to store elastic energy. However, at high shear rates due to fluid-like behavior of RBCs, viscoelastic effects are also less prominent. Therefore, viscoelastic models are more adequate for blood flow at low shear stress and in oscillatory flow conditions [26].

Motivated by above facts, we are interested to investigate the flow characteristics of blood in a narrow catheterized artery by considering blood as a two-phase fluid. The aggregate of red blood cells in the core region is modeled by Oldroyd-B fluid while the periphery region is assumed to behave as a Newtonian fluid. The compliant nature of the artery is not incorporated in the present study. A priori estimate about how the compliant nature of the artery wall may affect the flow rate, stability etc may be difficult to obtain. However, the interested reader are referred to reference [27] for further details. The layout of the paper is as follows: Fundamental laws and geometry of pulsatile flow problem are described in section 2. The governing equations of the problem and appropriate boundary conditions are derived in section 3. Solution obtained via Laplace transform is presented in section 4. Numerical solution through finite difference method is obtained in section 5. Section 6 comprises results and discussion for various values of parameters of interest. Finally, some conclusions are drawn in section 7.

## 2. Flow Equations

The continuity and momentum equations governing the flow of an incompressible fluid are

∇⋅u=0, (1)

$$\rho \frac{du}{dt}=\nabla \cdot T+\rho b,$$ (2)

where ***u*** is the fluid velocity, *ρ* is the density, ***T*** is the Cauchy stress, ***b*** is the body acceleration and *d*/*dt* is the material time derivative given by

$$\frac{d(\cdot )}{dt}=\frac{\partial (\cdot )}{\partial t}+\left(u\cdot \nabla \right).$$ (3)

The Cauchy stress tensor of an Oldroyd-B fluid is [26]

T=−pI+S, (4)

in which *p* is the pressure, ***I*** is the identity tensor and the extra stress tensor ***S*** satisfies the following expression

$$S+\lambda_{1}\frac{DS}{Dt}=\mu \left[A_{1}+\lambda_{2}\frac{DA_{1}}{Dt}\right],$$ (5)

where *λ*_1_, *λ*_2_ are the relaxation and retardation times respectively, *μ* is the co-efficient of viscosity, ***A***_1_ is the first Rivilin-Ericksen tensor and *D/Dt* is the contravariant convective derivative. The expression for *D/Dt* and ***A***_1_ are defined by

$$\frac{D(\cdot )}{Dt}=\frac{d(\cdot )}{dt}-L\left(\cdot \right)-\left(\cdot \right)L^{T},$$ (6)

$$\text{Where}\;A_{1}=L+L^{T},$$ (7)

L=∇u. (8)

## 3. Mathematical Formulation

**Fig 1:**
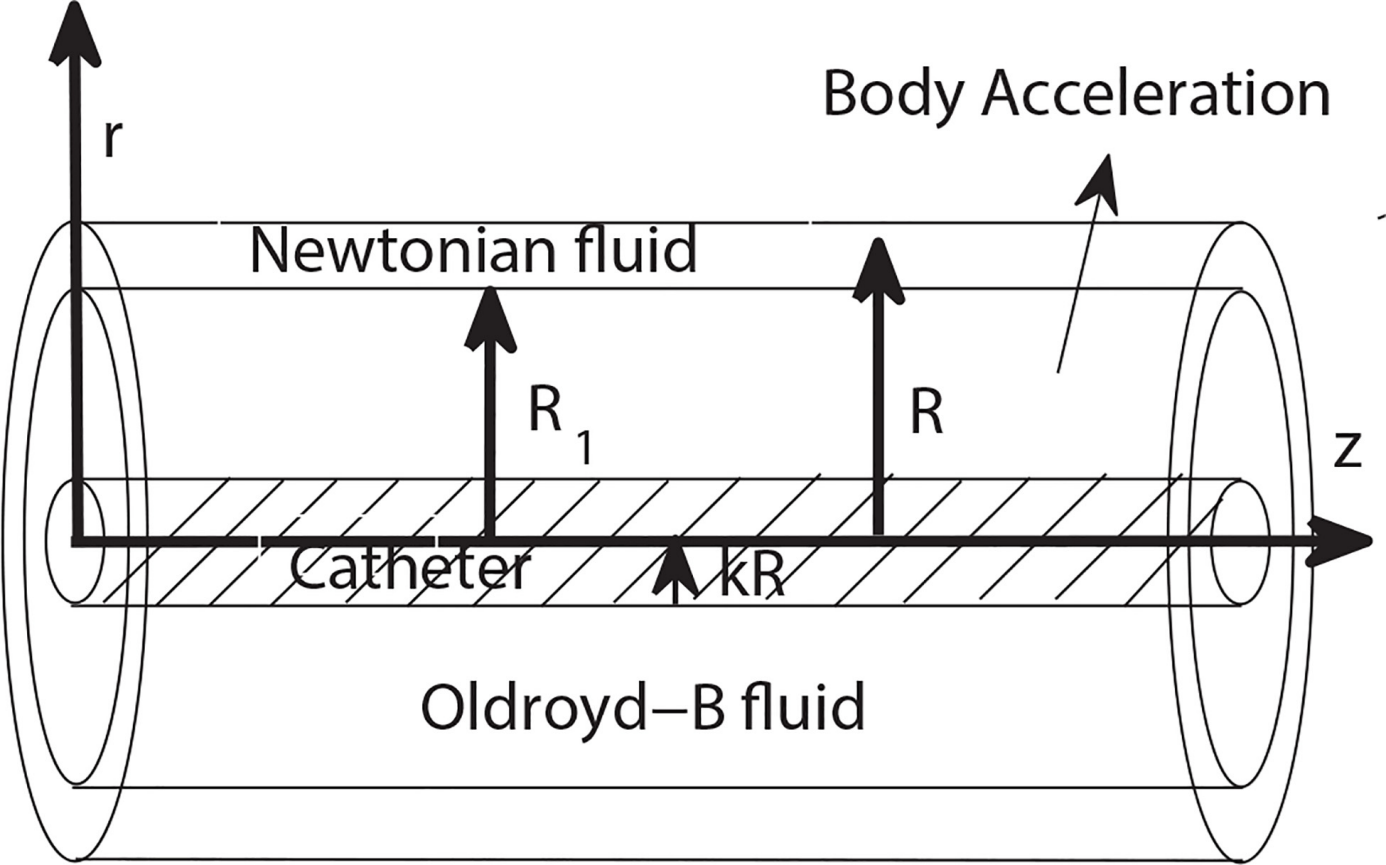
Geometry of the catheterized artery.

Consider an axially symmetric, unsteady, uni-directional, incompressible and two-phase flow of blood through an artery of radius *R* in which a catheter is inserted. The radius of catheter is *kR* (0 < *k* < 1). The blood in core and peripheral regions is modeled by Oldroyd-B fluid and Newtonian fluid, respectively. We use cylindrical coordinates (*r*,*θ*,*z*) to formulate the flow under consideration. Following [24], it is assumed that the flow is subject to periodic acceleration in the *z*-direction. The schematic diagram of the catheterized artery is presented in Fig 1.

Since the flow in the rigid catheterized tube is assumed to be unsteady and uni-directional, therefore we write velocity field in core and peripheral region as follows:

$$u_{i}=\left[0,0,u(r,t)\right],i=1,2.$$ (9)

The momentum equation in this case simplifies to

$$\rho \frac{\partial u}{\partial t}=-\frac{\partial p}{\partial z}+\rho G(t)+\frac{1}{r}\frac{\partial }{\partial r}\left(rS_{rz}\right),$$ (10)

where *G(t)* is periodic acceleration in *z*-direction. We divide our domain *kR* ≤ *r* ≤ *R* as *kR* ≤ *r* ≤ *R*_1_ (a core region) and *R*_1_ ≤ *r* ≤ *R* (a periphery region). For the present problem, the fluid in the core is an Oldroyd-B fluid while in the periphery region it is a Newtonian fluid. Thus the constitutive equation for the shear stress in periphery region is

$$S_{rz}=\mu_{2}\frac{\partial u_{2}}{\partial r},\;R_{1}\leq r\leq R,$$ (11)

where *μ*_2_ is the viscosity of the Newtonian fluid. The general form of the constitutive equation for shear stress in the core region is [26]

$$\left(1+\lambda_{1}\frac{\partial }{\partial t}\right)S_{rz}=\mu_{1}\left(1+\lambda_{2}\frac{\partial }{\partial t}\right)\frac{\partial u_{1}}{\partial r},\;kR\leq r\leq R_{1}.$$ (12)

The pressure gradient can be put in the form [28]:

$$-\frac{\partial p}{\partial z}=A_{0}+A_{1}\cos\omega_{p}t.$$ (13)

Here *A*_0_ is the systolic and *A*_1_ is the amplitude (diastolic) components of the pressure gradient, *ω*_*p*_ = 2*πf*_*p*_ is the circular frequency and *f*_*p*_ is the pulse rate frequency. Following [29], we can write.

$$G(t)=A_{g}\cos\left(\omega_{b}t+\phi \right),$$ (14)

in which *A*_*g*_ is the amplitude, *f*_*b*_ is the frequency (*ω*_*b*_ = 2*πf*_*b*_) and *ϕ* is the lead angle of *G*(*t*) with respect to the heart action. Eliminating *S*_*rz*_ between (10) and (12), we get in the core region

$$\begin{array}{l} \rho_{1}\left(\frac{\partial u_{1}}{\partial t}+\lambda_{1}\frac{\partial^{2}u_{1}}{\partial t^{2}}\right)=A_{0}\left(1+\frac{A_{1}}{A_{0}}cos(\omega_{p}t)-\lambda_{1}\frac{A_{1}}{A_{0}}\omega_{p}sin\left(\omega_{p}t\right)\right)+\rho_{1}A_{g}\left(cos\left(\omega_{b}t+\phi \right)-\lambda_{1}\omega_{b}sin\left(\omega_{b}t\right)\right)\\ \;+\mu_{1}\left(\frac{\partial^{2}u_{1}}{\partial r^{2}}+\lambda_{2}\frac{\partial^{3}u_{1}}{\partial r^{2}t}\right)+\frac{\mu_{1}}{r}\left(\frac{\partial u_{1}}{\partial r}+\lambda_{2}\frac{\partial^{2}u_{1}}{\partial rt}\right), \end{array}$$ (15)

and for the periphery region.

$$\rho_{2}\frac{\partial u_{2}}{\partial t}=A_{0}\left(1+\frac{A_{1}}{A_{0}}cos(\omega_{p}t)\right)+\rho_{2}A_{g}cos\left(\omega_{b}t+\phi \right)+\frac{\mu_{2}}{r}\frac{\partial u_{2}}{\partial r}+\mu_{2}\frac{\partial u_{2}}{\partial r^{2}},$$ (16)

where subscript denotes differentiation with respect to the indicated variable.

The boundary and initial conditions for the present flow situation are [26]:

$$\begin{array}{l} {u_{1}|}_{r=kR}=0,{u_{2}|}_{r=1}=0,{u_{1}|}_{r=R_{1}}={u_{2}|}_{r=R_{1}},\\ \mu_{2}\left(1+\lambda_{1}\frac{\partial }{\partial t}\right)\frac{\partial u_{2}}{\partial r}=\mu_{1}\left(1+\lambda_{2}\frac{\partial }{\partial t}\right)\frac{\partial u_{1}}{\partial r},\;at\;interface\;r=R_{1}, \end{array}$$

$$u_{1}=u_{2}=0,\;at\;t=0,$$ (17)

The volumetric Flow rate and shear stress at the wall of the tube are respectively given by

$$Q=2\pi \overset{R_{1}}{\underset{0}{\int }}u_{1}r\;dr+\overset{R}{\underset{R_{1}}{\int }}u_{2}r\;dr,$$

$$\tau_{s}=\mu_{2}{\left(\frac{\partial u_{2}}{\partial r}\right)}_{r=R}.$$ (18)

The resistance to flow or impedance experienced by flowing blood at any cross-section is [28]:

$$\Lambda =\frac{\left(\partial p/\partial z\right)}{Q}.$$ (19)

### 3.1. Dimensionless formulation

We are interested in numerical solution of Eqs 15 and 16 subject to conditions 17, for this we first normalize these equations by defining [29]

$$\bar{r}=\frac{r}{R},\;\bar{u}=\frac{u}{U_{0}},\;\bar{t}=\frac{\omega_{p}}{2\pi }t.$$ (20)

In terms of new variable the momentum equations after dropping the bars takes the following form

$$\begin{array}{l} \alpha \left(\frac{\partial u_{1}}{\partial t}+\lambda_{1}\frac{\partial^{2}u_{1}}{\partial t^{2}}\right)=B_{1}\left(1+e\;cos(2\pi t)\right)-\left(2\lambda_{1}eB_{1}\pi \right)sin\left(2\pi t\right)+B_{2}\left(cos\left(2\pi \omega_{r}t+\phi \right)\right)\\ \;-\left(2\pi \omega_{r}B_{2}\lambda_{1}\right)\sin\left(2\pi \omega_{r}t\right)+\left(\frac{\partial^{2}u_{1}}{\partial r^{2}}+\lambda_{2}\frac{\partial^{3}u_{1}}{\partial r^{2}t}\right)+\frac{1}{r}\left(\frac{\partial u_{1}}{\partial r}+\lambda_{2}\frac{\partial^{2}u_{1}}{\partial rt}\right), \end{array}$$ (21)

$$\gamma \frac{\partial u_{2}}{\partial t}=B_{1}\left(1+e\;cos(2\pi t)\right)+B_{2}\left(cos\left(2\pi \omega_{r}t+\phi \right)\right)+\frac{1}{r}\frac{\partial u_{2}}{\partial r}+\frac{\partial u_{2}}{\partial r^{2}}.$$ (22)

The dimensionless boundary and initial conditions become

$$\begin{array}{l} {u_{1}|}_{r=k}=0,\;{u_{2}|}_{r=1}=0,\;{u_{1}|}_{r=\beta }={u_{2}|}_{r=\beta },\;{u_{1}|}_{\left(r,0\right)}={u_{2}|}_{\left(r,0\right)}=0\\ \left(1+\lambda_{2}\frac{\partial }{\partial t}\right)\frac{\partial u_{1}}{\partial r}=\mu^{*}\left(1+\lambda_{1}\frac{\partial }{\partial t}\right)\frac{\partial u_{2}}{\partial r},\;at\;interface\;r=\beta , \end{array}$$ (23)

Similarly the volume flow rate and the shear stress in dimensionless form are

$$Q=2\pi R^{2}\left(\overset{\beta }{\underset{k}{\int }}u_{1}rdr+\overset{1}{\underset{\beta }{\int }}u_{2}rdr\right),$$ (24)

$$\tau_{s}=\frac{1}{R}{\left(\frac{\partial u_{2}}{\partial r}\right)}_{r=1}.$$ (25)

The various parameter appearing in Eqs 21–25 are defined as follows [28]:

$$U_{0}=\left(\frac{A_{0}R^{2}}{\mu_{1}}\right),\;{\bar{\lambda }}_{1}=\frac{\lambda_{1}\omega_{p}}{2\pi },\;{\bar{\lambda }}_{2}=\frac{\lambda_{2}\omega_{p}}{2\pi },\;e=\frac{A_{1}}{A_{0}},\;\omega_{r}=\frac{\omega_{b}}{\omega_{p}},$$

$$\rho^{*}=\frac{\rho_{2}}{\rho_{1}},\;\mu^{*}=\frac{\mu_{2}}{\mu_{1}},B_{1}=\frac{A_{0}R^{2}}{\mu_{1}\;U_{0}},\;B_{2}=\rho_{1}A_{g}\frac{R^{2}}{\mu_{1}\;U_{0}}=\frac{\rho_{1}A_{g}}{A_{0}}B_{1},\;\beta =\frac{R_{1}}{R},$$

$$\alpha =\frac{\rho_{1}\omega_{p}R^{2}}{2\pi \;\mu_{1}},\;\gamma =\frac{\rho_{2}\omega_{p}R^{2}}{2\pi \;\mu_{2}}=\frac{\rho_{1}\omega_{p}R^{2}}{2\pi \;\mu_{1}}\frac{\rho_{2}}{\mu^{*}\rho_{1}}=\alpha \frac{\rho^{*}}{\mu^{*}},{\bar{\tau }}_{s}=\frac{\tau_{s}}{\mu_{2}U_{0}},$$

$${\hat{B}}_{1}=\frac{A_{0}R^{2}}{\mu_{1}\;U_{0}\mu^{*}}=\frac{B_{1}}{\mu^{*}},\;{\hat{B}}_{2}=\frac{\rho_{2}A_{g}R^{2}}{\mu_{1}\;U_{0}\mu^{*}}=\frac{\rho_{1}A_{g}R^{2}}{\mu_{1}\;U_{0}\mu^{*}}=\rho_{1}A_{s}\frac{R^{2}}{\mu_{1}\;U_{0}}\frac{\rho_{2}}{\rho_{1}\mu^{*}}=B_{2}\frac{\rho^{*}}{\mu^{*}}.$$ (26)

## 4. Analytical Solution

Eqs 21 and 22 subject to the initial and boundary condition given in Eq 23 can be solved analytically by employing Laplace transform. To this end, let us denote *U*_1_(*r*,*s*) and *U*_2_(*r*,*s*), respectively, the Laplace transform of *u*_1_(*r*,*t*) and *u*_2_(*r*,*t*). Then Eqs 21 and 22 reduce to following ordinary differential equations in transformed domain

$$U_{1rr}+\frac{U_{1r}}{r}-\alpha \frac{\left(s+\lambda_{1}s^{2}\right)}{\left(1+\lambda_{2}s\right)}U_{1}=\Omega \left(s\right),$$ (27)

$$U_{2rr}+\frac{U_{2r}}{r}-\gamma sU_{2}=\beta \left(s\right),$$ (28)

where

$$\Omega \left(s\right)=\frac{1}{\left(1+s\lambda_{2}\right)}\left(\frac{C}{s^{2}+4\pi^{2}}-\frac{B_{1}}{s}-\frac{B}{s^{2}+4\pi^{2}\omega_{r}^{2}}\right),$$ (29)

$$B=2\pi B_{2}\omega_{r}sin\phi +4\pi^{2}B_{2}\lambda_{1}\omega_{r}^{2}-B_{2}cos\phi \;s,$$ (30)

$$C=4\pi^{2}B_{1}\lambda_{1}e-B_{1}e\;s.$$ (31)

$$\beta_{1}\left(s\right)=-\left(\frac{B_{1}}{s}-\frac{eB_{1}s}{s^{2}+4\pi^{2}}\right)+B_{2}\left(cos\phi s-sin\phi \;2\pi \;\omega_{r}\right)\frac{1}{s^{2}+4\pi^{2}\omega_{r}^{2}}.$$ (32)

The boundary and initial conditions in the transform domain read

$$U_{1}\left(k\right)=0,U_{2}\left(1\right)=0,$$ (33)

$$U_{1}\left(\beta_{1}\right)=U_{2}\left(\beta_{1}\right),$$ (34)

$$U_{2r}(\beta_{1})=\mu^{*}\left(\frac{1+s\lambda_{2}}{1+s\lambda_{1}}\right)U_{1r}(\beta_{1}).$$ (35)

The condition (35) is established by taking Laplace transform of both sides of interface condition in Eq 17 and using the *u*_1*t*_ = *u*_2*t*_ *at t* = 0. The differential Eqs 27 and 28 can be readily solved in terms of Bessel’s functions to get the expressions of *U*_1_(*r*,*s*) and *U*_2_(*r*,*s*) as

$$U_{1}\left(r,s\right)=-\frac{\Omega \left(s\right)\left(1+\lambda_{2}s\right)}{\alpha s\left(1+\lambda_{1}s\right)}+c_{1}I_{0}\left(r\sqrt{\alpha \;s\left(1+\lambda_{1}s\right)/\left(1+\lambda_{2}s\right)}\right)+c_{2}K_{0}\left(r\sqrt{\alpha \;s\left(1+\lambda_{1}s\right)/\left(1+\lambda_{2}s\right)}\right),$$ (36)

$$U_{2}\left(r,s\right)=-\frac{\beta_{1}\left(s\right)}{\gamma s}+c_{3}I_{0}\left(r\sqrt{\gamma \;s}\right)+c_{4}K_{0}\left(r\sqrt{\gamma s}\right).$$ (37)

The values of arbitrary constants *c*_1–4_ can be easily calculated by implementing the boundary conditions (33)–(35). Due to the complicated nature of the solution in transformed domain, analytical inversion is difficult. Therefore, we have relied on the numerical inversion using Mathematica Package *NumericalInversion*.*m*. The command “*Stehfest*” is used to get the numerical values of the inverted solution.

## 5. Numerical Solution

Eqs 21 and 22 subject to the boundary condition given in Eq 23 are solved numerically using the finite difference method [30, 31]. The uniformly distributed discrete points in radial direction are defined as *r*_*i*_ = (*i*−1)Δ*r*, (*i* = 1,2,….,*N*_*c*_ + 1) such that $r_{\left(N_{c}+1\right)}=\beta$ and *r*_*i*_ = (*i*−(*N*_*c*_+1))Δ*r*, *i* = (*N*_*c*_ + 1,*N*_*c*_ + 2,…,*N* + 1) such that *r*_*N*+1_ = 1, where Δ*r* is the increment in the radial direction. Similarly we define *t*_*j*_ = (*j*−1)Δ*t*, (*j* = 1,2,….) as discrete time points with Δ*t* indicating the small time increment. For problem under consideration we have chosen Δ*r* = 0.025 and Δ*t* = 0.0001. This choice of Δ*r* and Δ*t* yields results convergent up to order 10^−7^. Let us denote the discretized value of *u*_*k*_ (*k* = 1,2) at (*r*_*i*_,*t*_*j*_) by $u_{k\;i}^{j}$. In this notation, finite difference formulas for first and second order derivatives read

$$\frac{\partial u_{k}}{\partial r}≅\frac{u_{k\;i+1}^{j}-u_{k\;i-1}^{j}}{2\;\Delta r}=u_{k\;r},$$

$$\frac{\partial^{2}u_{k}}{\partial r^{2}}≅\frac{u_{k\;i+1}^{j}-2u_{k\;i}^{j}+u_{k\;i-1}^{j}}{{\left(\Delta r\right)}^{2}}=u_{k\;r^{2}}.$$ (38)

For the time derivative, we define the approximation:

$$\frac{\partial u_{k}}{\partial t}≅\frac{u_{k\;i}^{j+1}-u_{k\;i}^{j}}{\Delta t}=u_{k\;t},$$

$$\frac{\partial^{2}u_{k\;i}}{\partial t^{2}}=\frac{u_{k\;i}^{j+1}-2u_{k\;i}^{j}+u_{k\;i}^{j-1}}{{\left(\Delta t\right)}^{2}}=u_{k\;t^{2}},$$

$$\frac{\partial }{\partial t}\left(\frac{\partial u_{k}}{\partial r}\right)≅\frac{u_{k\;i+1}^{j+1}-u_{k\;i+1}^{j}-u_{k\;i-1}^{j+1}+u_{k\;i-1}^{j}}{2\;\Delta r\;\Delta t}=u_{k\;tr},$$

$$\frac{\partial }{\partial t}\left(\frac{\partial^{2}u_{k}}{\partial r^{2}}\right)≅\frac{u_{k\;i+1}^{j+1}-2u_{k\;i}^{j+1}+u_{k\;i-1}^{j+1}-u_{k\;i+1}^{j}+2u_{k\;i}^{j}-u_{k\;i-1}^{j}}{{\left(\Delta r\right)}^{2}\Delta t}=u_{k\;tr^{2}}.$$ (39)

Utilizing (38) and (39), Eqs 21 and 22 may be transformed to the following difference equations:

$$\begin{array}{l} u_{1}{}_{i}^{j+1}=\frac{1}{\left(1+\frac{\lambda_{1}}{\Delta t}\right)}\left[u_{1}{}_{i}^{k}\left(1+\frac{2\lambda_{1}}{\Delta t}\right)-\frac{\lambda_{1}}{\Delta t}u_{1}{}_{i}^{j-1}\right]+\left(\frac{\Delta t}{\alpha }\right)[B_{1}\left(1+ecos(2\pi t^{j})\right)-\left(2\lambda_{1}eB_{1}\pi \right)\sin\left(2\pi t^{j}\right)\\ \;+B_{2}\left(\cos\left(2\pi \omega_{r}t^{k}+\phi \right)\right)-\left(2\pi \omega_{r}B_{2}\lambda_{1}\right)\sin\left(2\pi \omega_{r}t^{j}\right)+\left\{u_{1_{r^{2}}}+\lambda_{2}u_{1_{{tr}^{2}}}\right\}+\left\{\frac{1}{r}\left(u_{1}{}_{r}\right)+\frac{\lambda_{2}}{r}u_{1}{}_{tr}\right\}], \end{array}$$ (40)

$$u_{2}{}_{i}^{j+1}=u_{2}{}_{i}^{j}+\left(\frac{\Delta t}{\gamma }\right)\left[{\hat{B}}_{1}\left(1+ecos\left(2\pi t^{j}\right)\right)+{\hat{B}}_{2}\left(\cos\left(2\pi \omega_{r}t^{j}+\phi \right)\right)+\frac{1}{r}\left[u_{2}{}_{r}\right]+u_{2_{r^{2}}}\right].$$ (41)

The boundary and initial conditions in the discretize form can be written as follows:

$$\begin{array}{l} u_{11}^{j}=u_{21}^{j},u_{2N+1}^{j}=0,u_{1N_{c}+1}^{j}=u_{2N_{c}+1}^{j},u_{1i}^{1}=u_{2i}^{1}=0,\\ u_{2kr}+\lambda_{1}u_{2ktr}=\mu^{*}\left(u_{1}{}_{kr}+\lambda_{1}u_{1}{}_{ktr}\right),\;at\;N_{c}+1, \end{array}$$ (42)

The partial differential equations governing the flow are solved both numerically and analytically. The details of the stability of numerical scheme used by us are given in the book by Hoffman, in which it is mentioned that the stability of explicit finite scheme depends on time and spatial step sizes. The numerical solution obtained by us fulfill all these requirements on spatial and temporal step sizes. Following Hoffman [32], we have chosen Δ*x* = 0.025, Δ*t* = 0.0001. This choice of Δ*x* and Δ*t* yield results convergent up to order 10^−7^. However it is observed during the computations that the numerical results are much sensitive to the choice of involved parameters. Thus for the present problem the stability of the numerical solution cannot be guaranteed only by full filling the requirements of the temporal and spatial step sizes. In fact with the present choice of spatial and temporal step sizes a stable numerical solution is possible only if material parameters *λ*_1_ and *λ*_2_ are such that 0.1 < *λ*_1_ < 0.5 and 0.1 < *λ*_2_ < 0.5.

## 6. Results and Discussion

In this section, we are interested to analyze the graphical results of velocity, flow rate, wall shear stress and resistance to flow results for different values of non-dimensional variables namely the catheter radius ratio (*k*), amplitude of pulsatile pressure gradient (*e*), relaxation and retardation time (*λ*_1_ and *λ*_2_) and generalized Womersley frequency parameter (*α*). In the present analysis the height of interface is assumed independent of the involved parameters and therefore it is not determined as a part of the problem. Instead we have taken it as a constant. The primary motivation for this assumption is based the evidence that RBC is migrate away from the wall leaving a cell-depleted layer near the wall. In such situation one can model blood flow in two stages (i) a peripheral layer modeled as a Newtonian liquid and (ii) a core region modeled as a non-Newtonian fluid. The thickness of both layer is assumed constant. Based on this fact several authors for instance Maji and Nair [33], Massoudi and Phouc [29], Ikbal et al. [31], Bugliarello and sevilla [34], Sulkla et al. [35], Akay an Kaye [36], Pralhad and Schultz [37], Sajid et al. [38] and Srivastava and Sexena [39] have used two-layered fluid model with constant peripheral thickness to study blood flow through arteries. We have followed these studies in our analysis. However, we have also shown the results for different interface positions in Figures (4, 10 and 11). The physiologically relevant values of the above mentioned parameters is listed in Tables 1 and 2.

**Table 1:** Plausible values of involved parameters for different blood vessels [29, 40, 41].

Parameters	Arterioles	Coronary artery	Femoral artery	Capillaries
*R(mm)*	0.685	1.5	5	0.24
*B*_1_	1.41	1.41	6.6	6.6
*B*_2_	2.07	21.67	4.64	3.4
*f*_*p*_	1.2	1.2	1.2	2.4
*f*_*g*_	1.2	1.2	1.3	1.2
*A*_0_ (Pa/mm)	7	698.5	32	20
*ρ* (Kg/m^3^)	1050	1050	1050	1050

**Table 2:** Values of Womersley number for different blood vessels [42].

Blood vessel	Diameter(mm)	*α*
Femoral artery	12.9	3.5
Arterioles	1.37	0.04
Capillaries	0.48	0.05

The radius ratio *k*, characterizing the radius of the catheter is assumed to be in the range *0*.*1–0*.*5*. The length of separation point between peripheral layer and the core region is represented by *β*. In this way, we get the thickness of the peripheral layer as *1*−*β*. In the present analysis, *β* are taken as 0.9.

**Fig 2:**
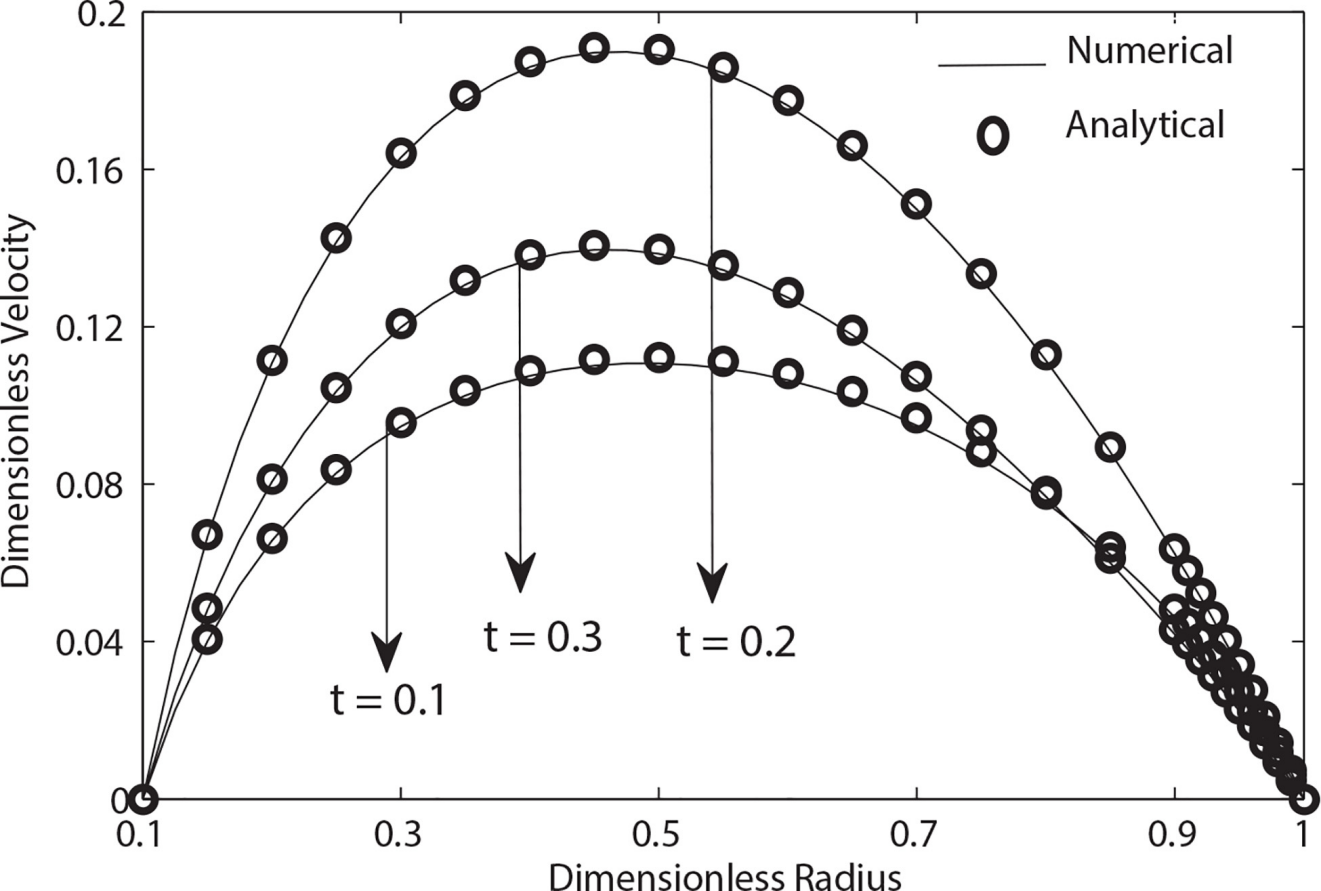
Variation of velocity with radial distance for (*α* = 0.5, *β* = 0.9, B_1_ = 2, B_2_ = 1, k = 0.1, e = 0.3).

**Fig 2**illustrates the profiles of velocity at different instants of time within a single cardiac cycle calculated through both numerical and analytical solutions. A pleasing agreement between both solutions is observed. This agreement also demonstrates the validity of our numerical scheme. Fig 2 further indicates an increase in velocity when *t* increases from 0.1 to 0.2 in systolic phase. However in diastolic phase i.e. at *t = 0*.*3* a decreasing trend in velocity is observed. The increasing behavior of velocity in systolic phase while opposite trend in diastolic phase is clearly due to the pulsatile pressure gradient produced by the pumping action of heart.

**Fig 3:**
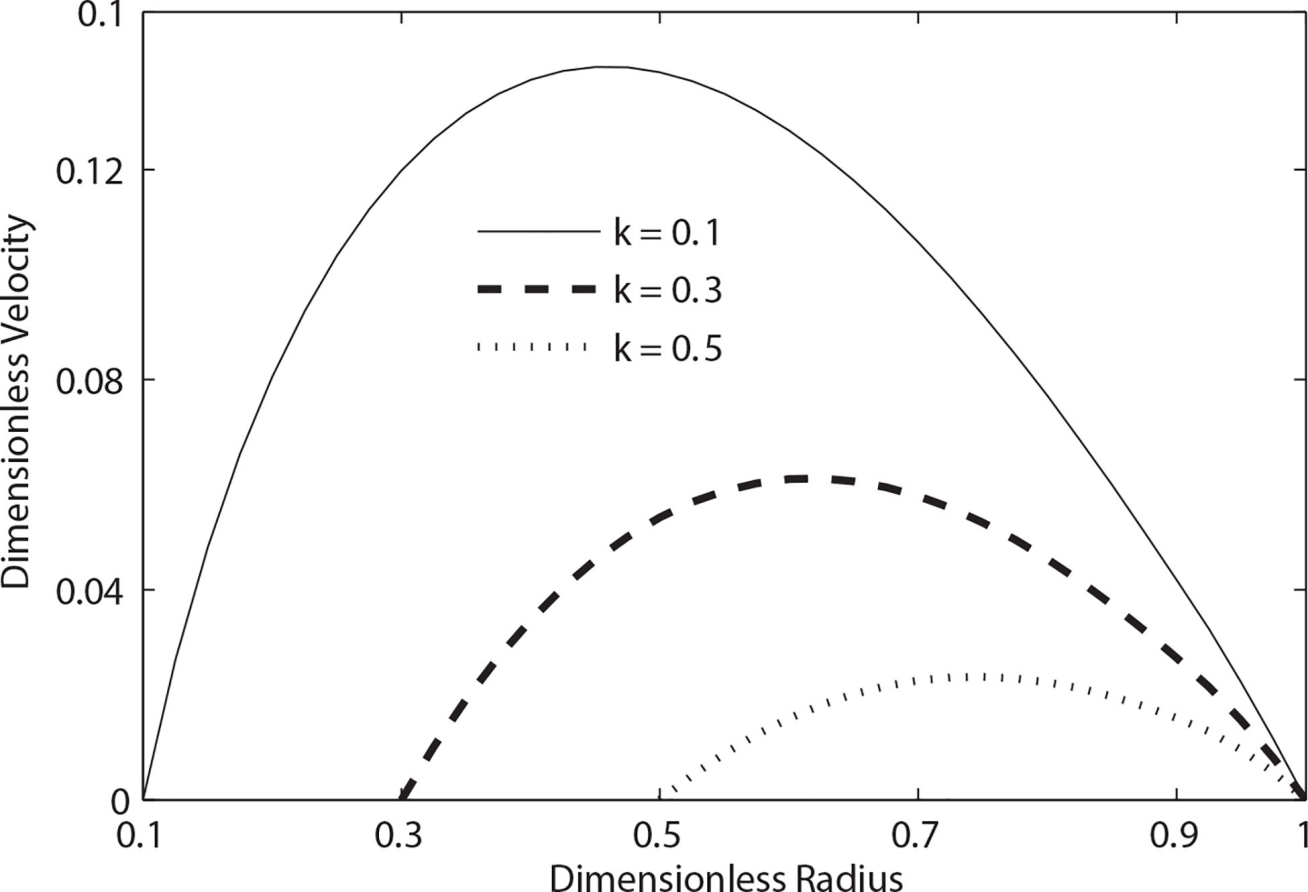
Variation of velocity with radial distance for different values of catheter radius (*t* = 0.3, *λ*_1_ = 0.2, *λ*_2_ = 0.1, *α* = 0.5, *β* = 0.9, B_1_ = 2, B_2_ = 1, e = 0.3).

**Fig 3**shows the effects of the catheterization at *t = 0*.*3* on the velocity distribution in the two-fluid model of blood flow. It is noticed that at a given instant of time *t = 0*.*3* with *β = 0*.*9* and the increasing values of the catheter radius ratio *k*, the velocity decreases considerably. It means that due to the insertion of catheter, the magnitude of the velocity reduces significantly.

**Fig 4:**
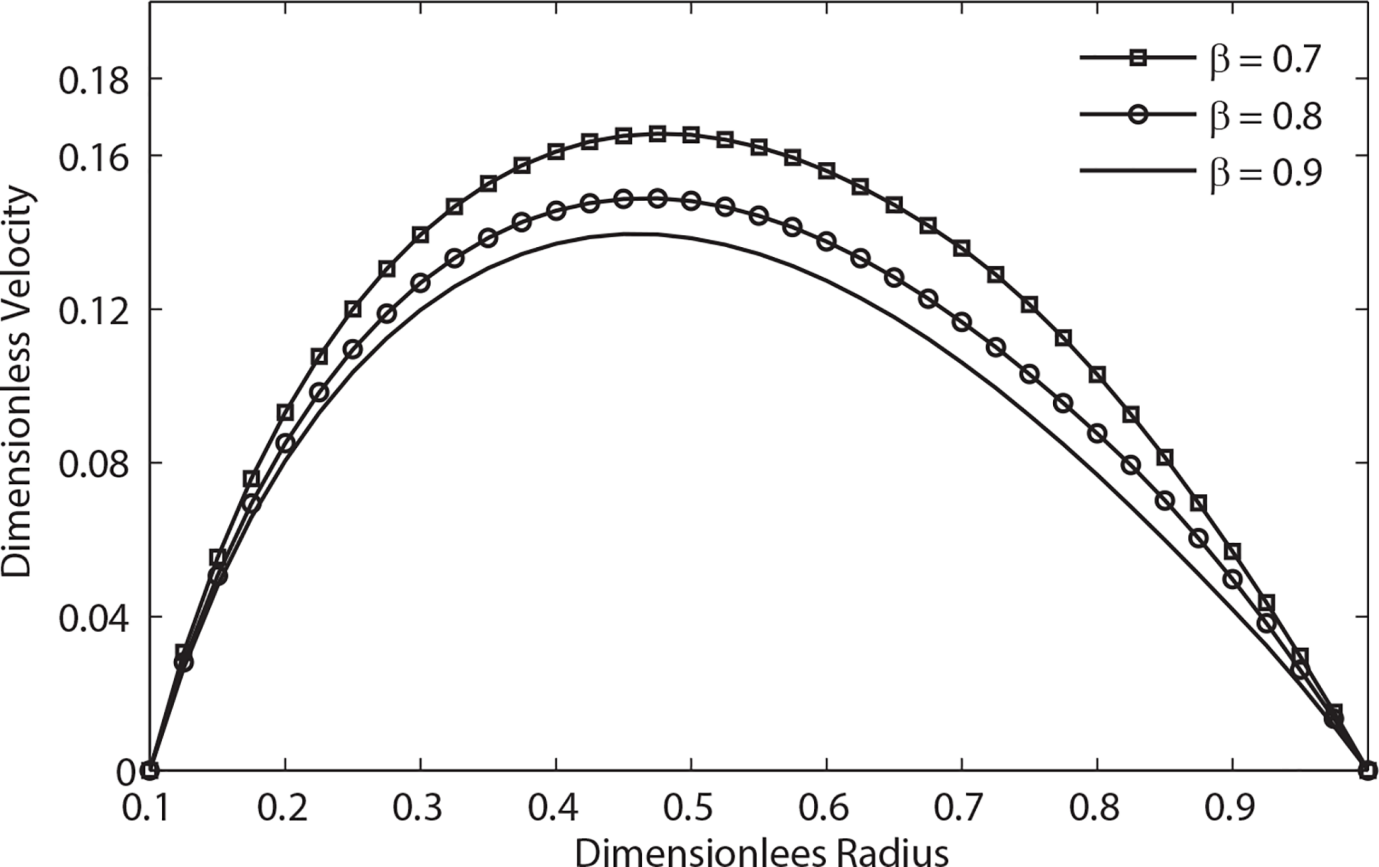
Variation of velocity with radial distance with different peripheral layer position (*t* = 1, e = 0.3, *λ*_1_ = 0.2, *λ*_2_ = 0.1, *α* = 0.5, B_1_ = 2, B_1_ = 1).

The velocity distribution for different values of the interface position *β* is shown in **Fig 4**for *k = 0*.*1*. It is found that for a given value of *k*, the velocity increases significantly with decreasing *β*, whereas the behavior is opposite when the width of the peripheral layer decreases (i.e., when the value of *β* decreases).

**Fig 5:**
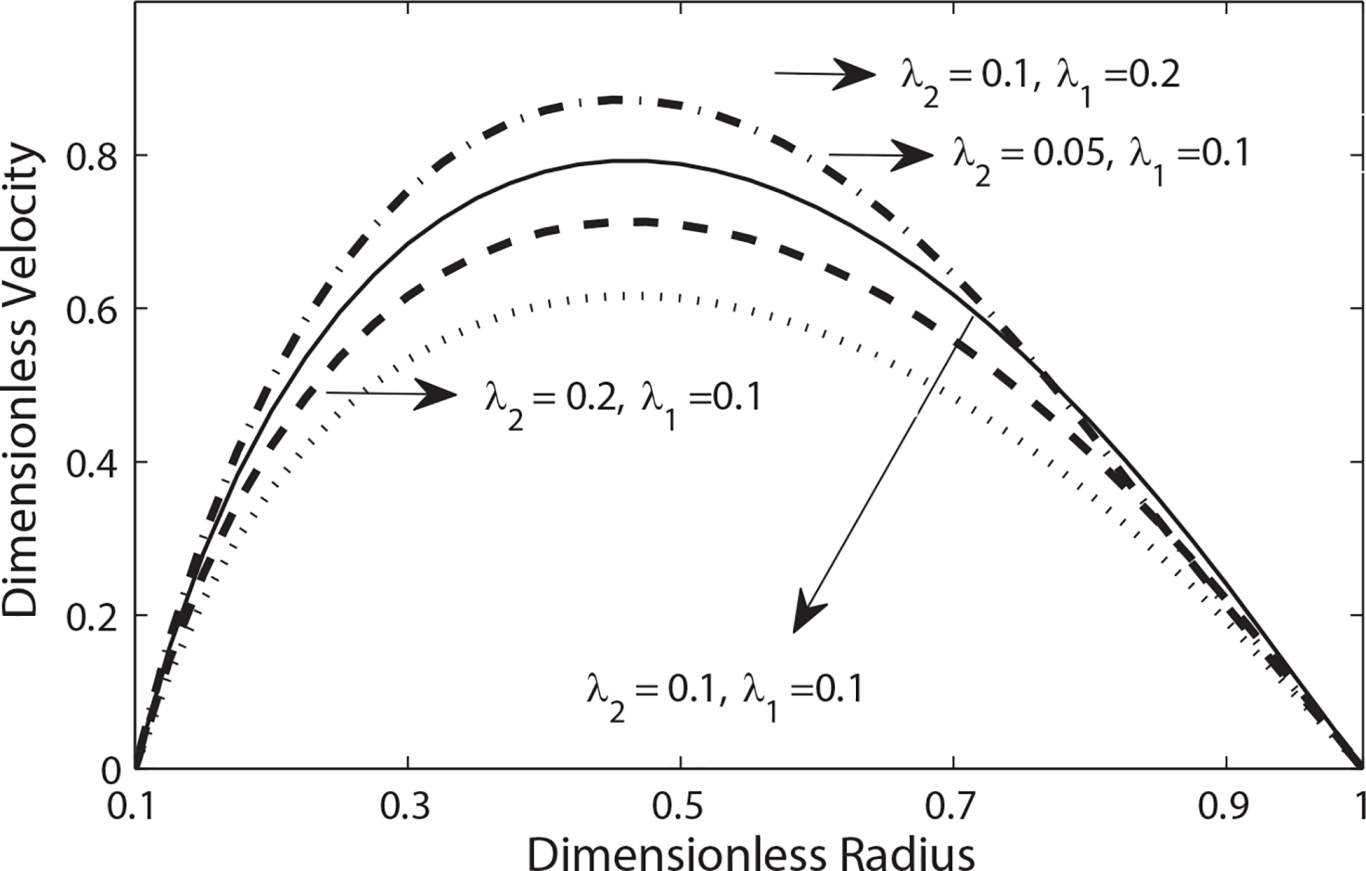
Variation of velocity with radial distance with (*t* = 0.3, B_2_ = 2, e = 0.5, *α* = 0.5, B_1_ = 2 *β* = 0.9).

We have shown the effects of relaxation and retardation times on the velocity profile in **Fig 5**. It is readily observed that the role of λ_1_ here is to increase the magnitude of velocity while λ_2_ counters the effects of λ_1_ and it decreases the magnitude of velocity.

**Fig 6:**
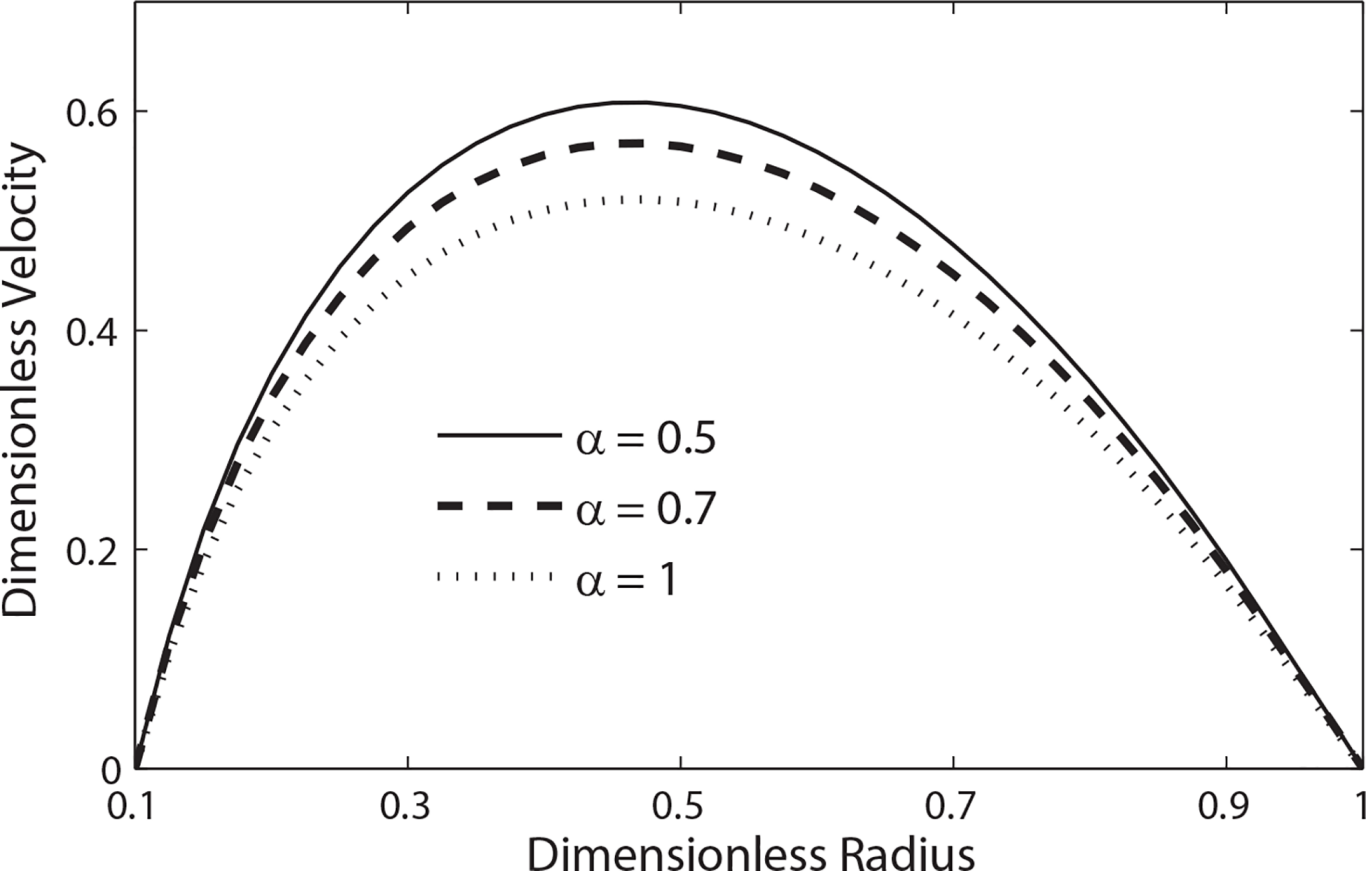
Variation of velocity with radial distance with the variation of *α* (*t* = 0.3, B_1_ = 2, *λ*_1_ = 0.2, *λ*_2_ = 0.2, e = 0.5, B_2_ = 2, *β* = 0.9).

**Fig 7:**
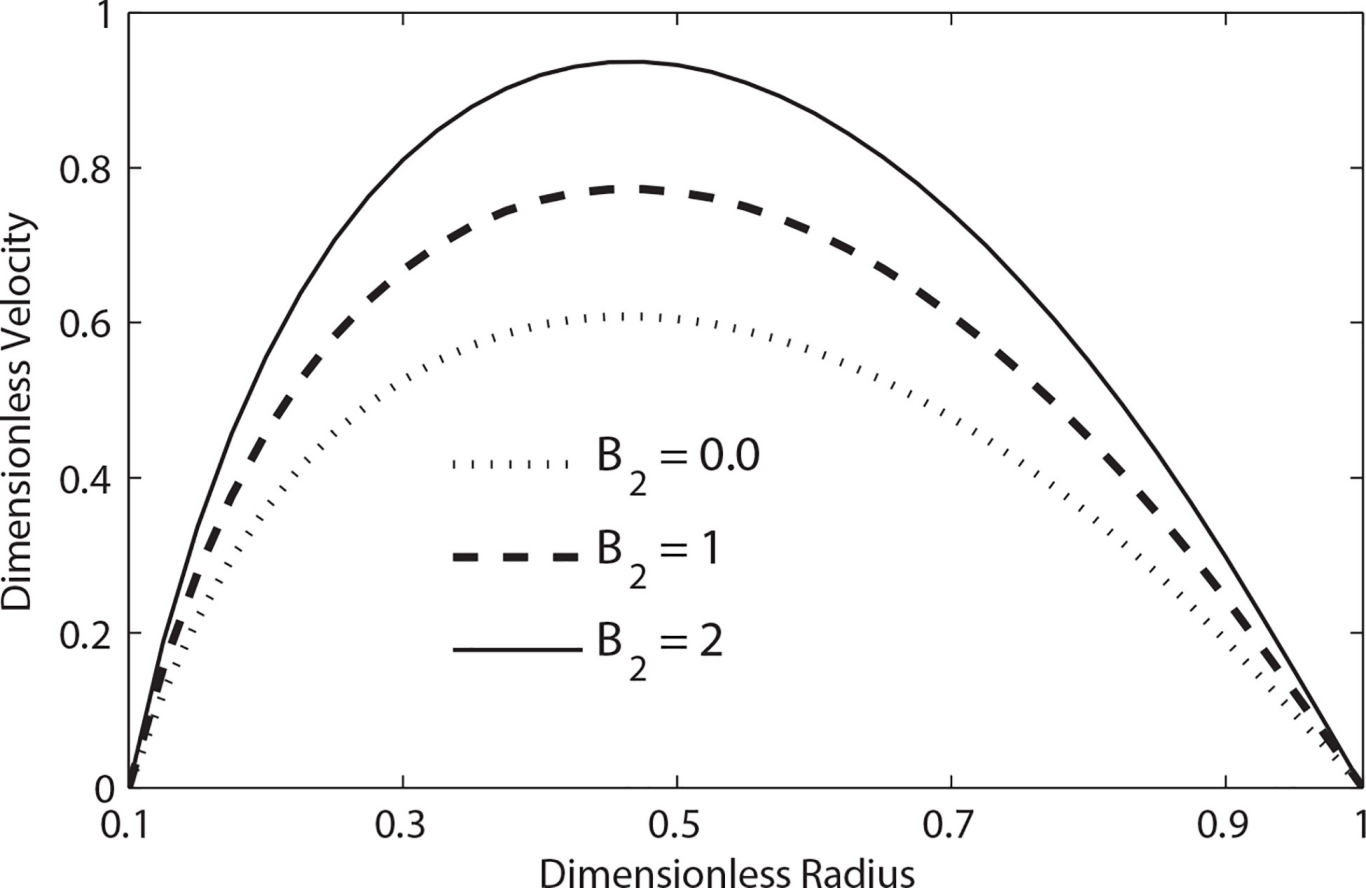
Variation of velocity with radial distance with the variation of *B*_2_ (*t* = 0.3, B_1_ = 2, *λ*_1_ = 0.2, *λ*_2_ = 0.2, e = 0.5, *β* = 0.9, *α* = 0.5).

**Fig 6**shows the variation of dimensionless velocity for different values of Womersley number *α* when *β = 0*.*9*, *t = 0*.*3* and *e = 0*.*5*. It is seen that at a given time instant the magnitude of velocity increases as the value of Womersley number decreases. **Fig 7**shows the velocity profile of blood for different values of body acceleration. It is evident from this figure that the magnitude of the velocity increases with the increase of amplitude of body acceleration.

**Fig 8:**
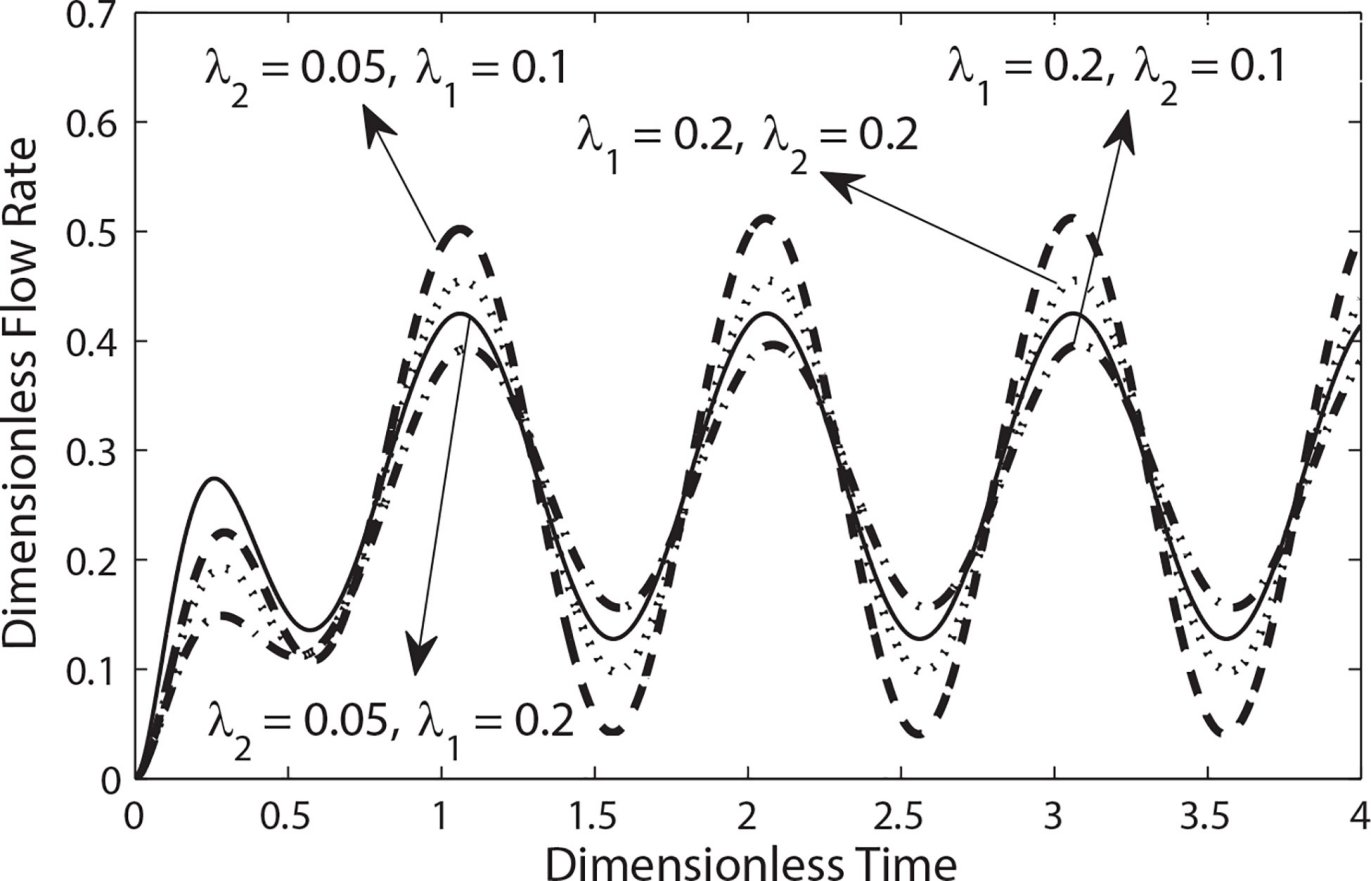
Variation of flow rate in a cycle of oscillation for different values of *λ*_1_ and *λ*_2_ with (*β* = 0.9, B_1_ = 2, e = 0.5, B_2_ = 0.0, *α* = 0.5).

**Fig 9:**
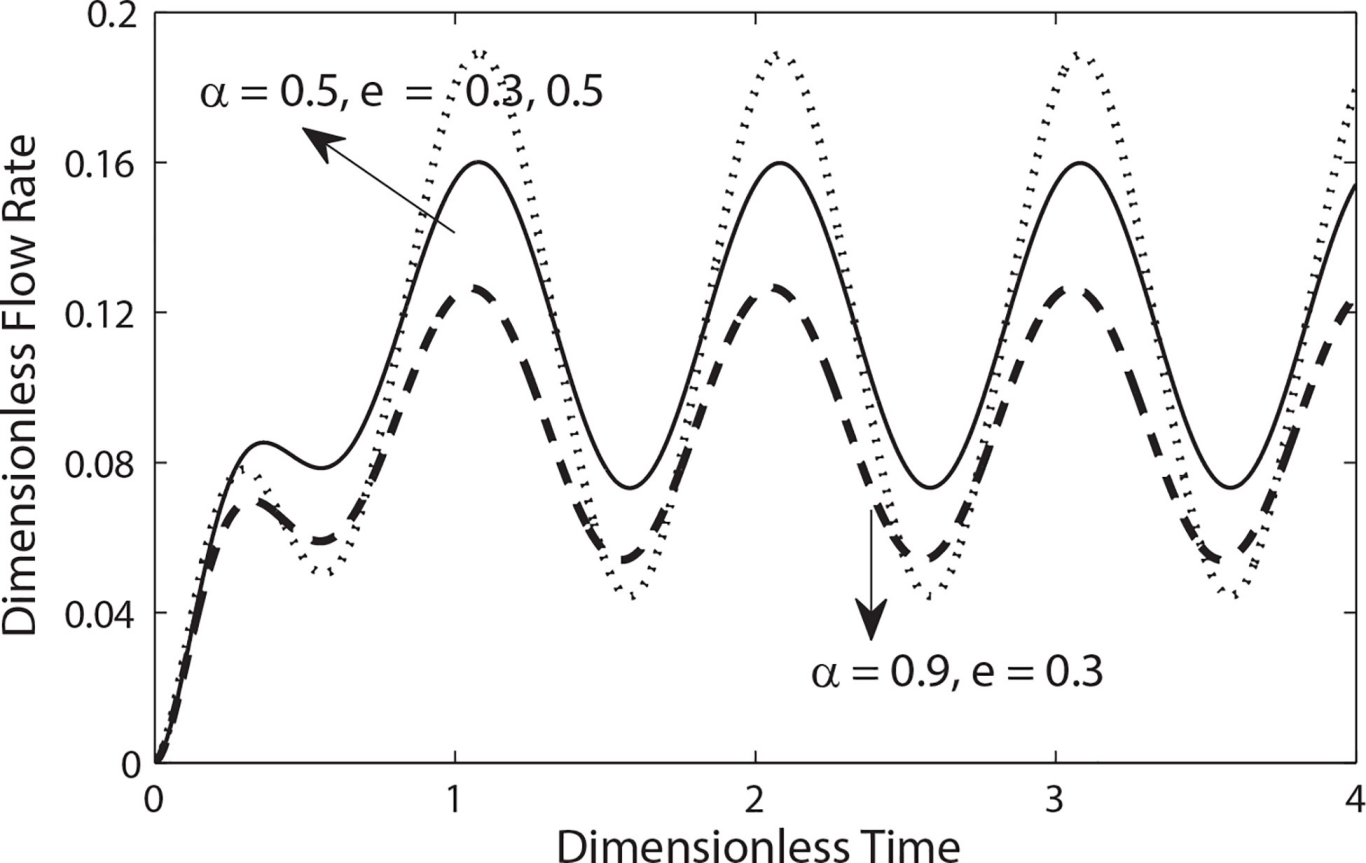
Variation of flow rate in a cycle of oscillation for different values of *e* and *α* with (e = 0.5, *λ*_1_ = 0.2, *λ*_2_ = 0.2, *β* = 0.9, k = 0.3, B_2_ = 2, B_2_ = 1).

**Fig 8**depicts the variation of flow rate for different values of *λ*_1_ and *λ*_2_ when *k = 0*.*1*, *e = 0*.*5 and β = 0*.*9*. Generally it is clear that the flow rate increases as *t* increases from 0 to 2.3 and then achieves its steady state condition. It means that flow rate fluctuates around its mean value with constant frequency and amplitude after achieving steady state condition. It is further observed from this figure that the amplitude of oscillations in flow rate increases (decreases) by increasing *λ*_1_(*λ*_2_). Similarly as expected that amplitude of oscillations in flow rate increases by increasing the amplitude of pulsatile pressure gradient (**Fig 9**). Moreover it also evident from Fig 9 that oscillation in flow rate diminishes by increasing Womersley number.

**Fig 10:**
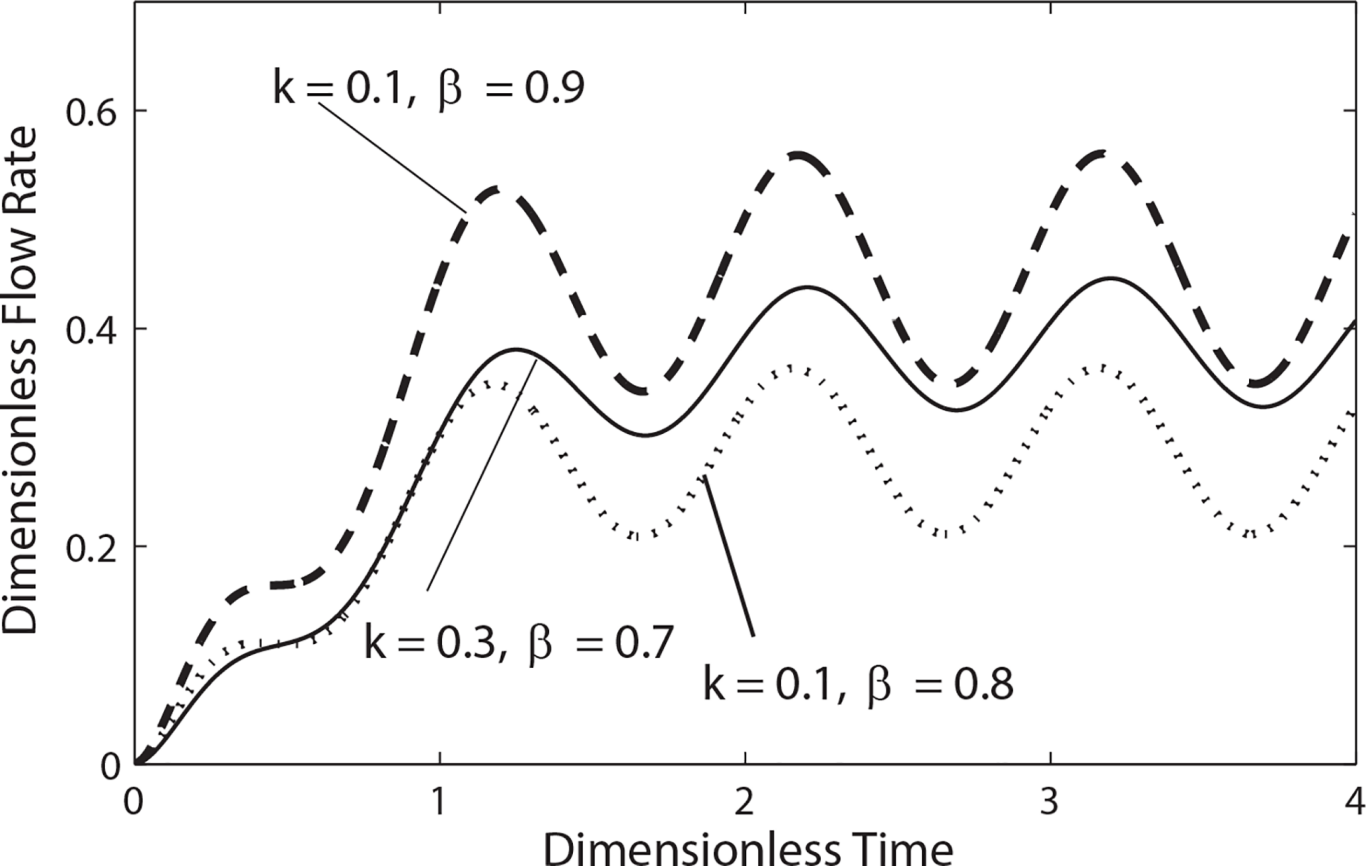
Variation of flow rate in a cycle of oscillation for different values of *k* and *β* with (e = 0.5, *λ*_1_ = 0.2, *λ*_2_ = 0.1, α = 0.3, B_1_ = 2, B_1_ = 1).

Similarly, the variation of dimensionless flow rate for different values of catheter radius ratio *k* and interface position *β* is shown in **Fig 10**. It is noticed that the flow rate decreases by increasing both catheter radius and peripheral layer thickness.

**Fig 11:**
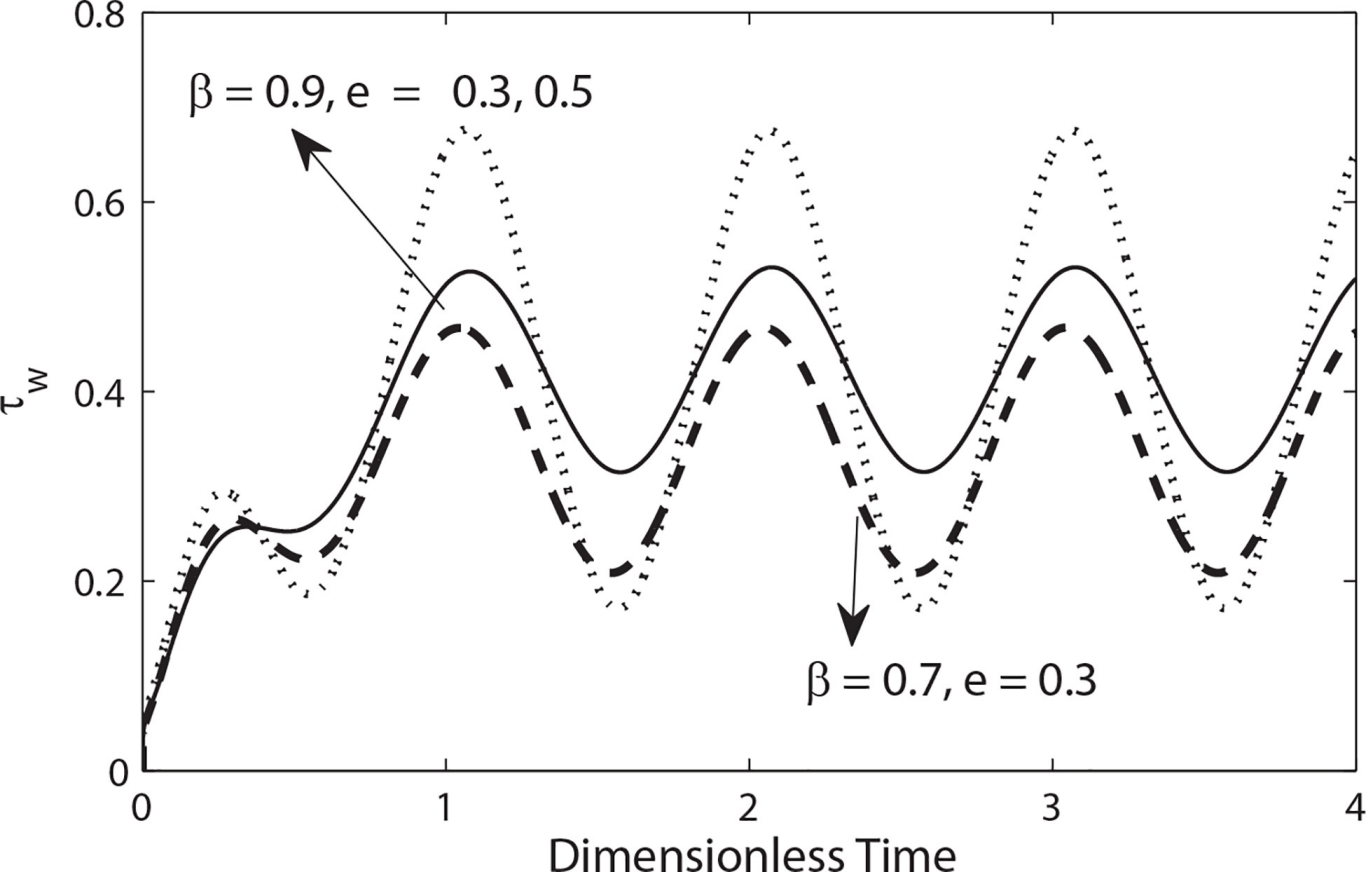
Variation of wall shear stress in a cycle of oscillation for different values of *e* and *β* with (e = 0.5, *λ*_1_ = 0.2, *λ*_2_ = 0.1, α = 0.3, k = 0.3, B_2_ = 1, B_1_ = 2).

In pulsatile flow, the non-dimensional wall shear stress *τ*_*w*_ can be calculated from Eq 25. The variation of wall shear stress in a cycle of oscillation for different values of amplitude *e* and peripheral thickness *β* with *k = 0*.*5* is shown in **Fig 11**. The wall shear stress fluctuates around its mean value as time increases. It is also noticed that amplitude of the wall shear is proportional to the magnitude of *e*. Its value increases with the increase in the magnitude of *e* while it shows opposite trend with the increase of peripheral radius length *β*.

**Fig 12:**
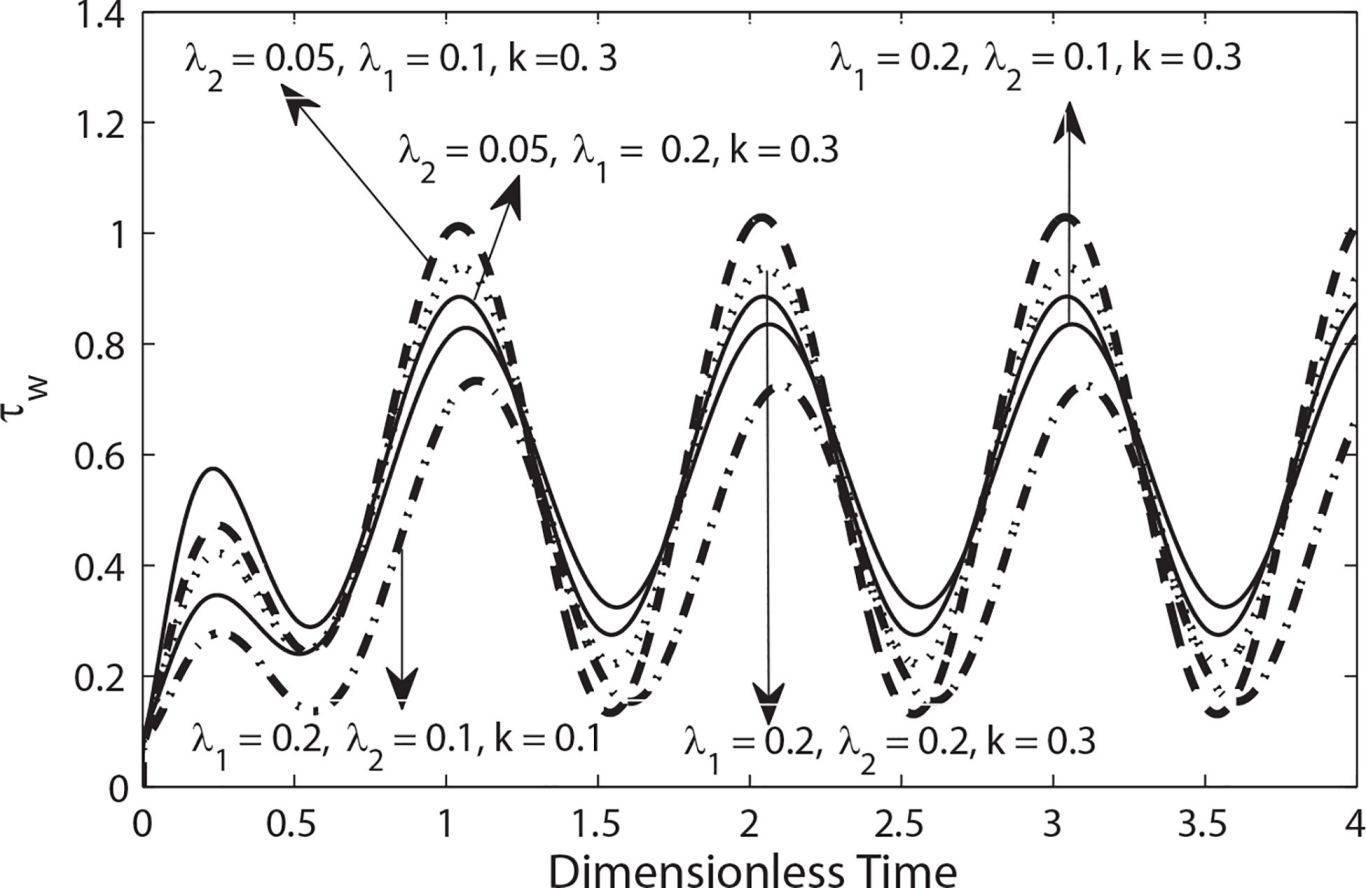
Variation of wall shear stress in a cycle of oscillation for different values of *k*, *λ*_2_ and *λ*_1_ (*β* = 0.9, e = 0.5, B_1_ = 2, *B*_2_ = 1.0, *α* = 0.5).

**Fig 12**shows the variation of wall shear stress in a cycle of oscillation for different values of *λ*_1_, *λ*_2_ and *k* with *β = 0*.*9*. This figure indicates that the magnitude of wall shear stress decreases by increasing *λ*_1_. while it is suppressed by increasing *λ*_2_. Moreover it is also observed through Fig 12 that the magnitude of the wall shear increases with an increase in the radius of catheter.

**Fig 13:**
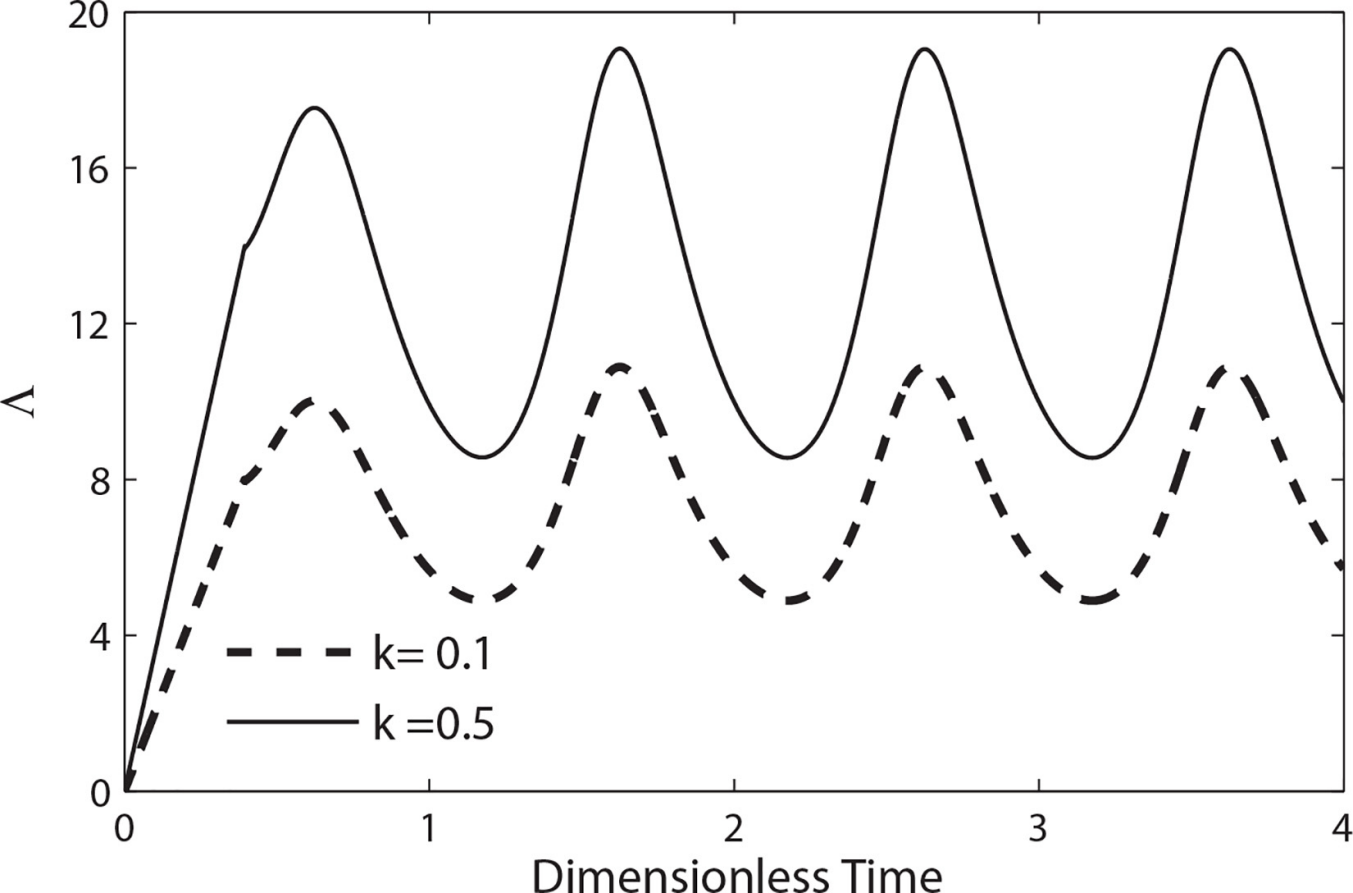
Variation of longitudinal impedance in a cycle of oscillation for different values of *k* with (*β* = 0.9, e = 0.1, B_2_ = 0, *λ*_1_ = 0.2, *λ*_2_ = 0.1, *α* = 0.3).

The longitudinal impedance (Λ) of the artery is calculated using Eq 19 and its variation during a flow cycle for different values of amplitude *k* with *e = 0*.*5* and *β = 0*.*9* is illustrated in **Fig 13**. It is observed that these profiles follow an opposite trend as compare to the flow rate profiles which is expected from the formula of resistance to flow given by expression (19). It is concluded from these profiles that the magnitude of resistance to flow increases with the increase of the size of catheter.

## 7. Conclusion

Two-layer mathematical model is developed for pulsatile flow of blood with the effects of body acceleration through a catheterized artery using Oldroyd-B fluid model in the core region and Newtonian model in the peripheral region. The analysis is based on the solution of a linear partial differential equation corresponding to each region. Assuming the continuity of velocity and no-slip conditions, the governing equation are solved in each region by employing finite difference technique. The validity of numerical solution is checked by comparing it with the analytical solution obtained through Laplace transform. An excellent agreement between both solutions is observed. After validation, numerical solution is further utilized to obtain various flow quantities. It is found that the resistance to flow or impendence, velocity, flow rate and wall shear stress are greatly affected by blood rheology, catheter radius and amplitude of pulsatile pressure gradient. More precisely a small increment in the catheter radius significantly decreases the magnitude of blood velocity and as a result of that a decrement in the flow rate is observed. As a consequence of decrease in velocity the wall shear stress and resistance to flow also increase considerably. Another important observation is that such quantitative measurement varies in magnitude by changing the rheological parameters of blood. In fact for a given catheter radius the flow rate of blood increases by increasing dimensionless relaxation time and as a result the resistance to flow decreases. Such observation may have certain implications for instance, the rheology of blood can be controlled to maintain the same flow in an artery with catheter as that in an artery without catheter. The results obtained through present computations may be related to hypertension. In normal circumstances/conditions, the amplitude of pressure gradient maintain the sufficient flow of blood in arteries for normal function of the organs. However, in situation where the person/human is subject to periodic body acceleration the results are quite different. In such situation the superimposed periodic oscillation due to body acceleration increase the amplitude of flow rate produced by pulsatile pressure gradient. This increase in the amplitude of the flow rate may results in hypertension, nausea, loss of vision and headache. On the contrary, the magnitude of the flow rate decreases with increasing the catheter radius. In such situation the impedance/ resistance to flow rate increases resulting in hypotension. Although the significance of the present study is limited but still this study throws some light on the flow behavior of blood. In fact this study presents the effects interface position, non-Newtonian nature and an inserted catheter on flow features. Analytical as well a numerical solution is also presented in this study. The analytical solution may be used as a benchmarking for more sophisticated 3D CFD analysis accounting pointing by the worthy reviewer.

## References

[1] Back L. H., “Estimated mean flow resistance increase during coronary artery catheterization”, Journal of Biomechanics, 27, pp.169–175 (1994). 8132684 10.1016/0021-9290(94)90205-4

[2] Back L. H., Denton T. A., “Some arterial wall shear stress estimates in coronary angioplasty”, Advanced Biological Engineering, 22, pp. 337–340 (1992).

[3] MacDonald D. A., “Pulsatile flow in a catheterized artery”, Journal of Biomechanics, 19, pp. 239 (1986). 3700436 10.1016/0021-9290(86)90156-9

[4] Karahalios G. T., “Some possible effects of a catheter on the arterial wall", Medical Physics, 17, pp. 922 (1990). 2233580 10.1118/1.596448

[5] Jayaraman G., Tiwari K., “Flow in a catheterized curved artery”, Medical & Biological Engineering & Computing, 33 (1995). 10.1007/BF02510793 8523917

[6] Dash R. K., Jayaraman G., Metha K. N., “Estimation of increased flow resistance in a narrow catheterized artery–A theoretical model”, Journal of Biomechanics, 29, pp. 917–930 (1996). 8809622 10.1016/0021-9290(95)00153-0

[7] Sankar D. S., Hemalatha K., “Pulsatile flow of Herschel–Bulkley fluid through catheterized arteries–A mathematical model”, Applied Mathematical Modeling, 31, pp.1497–1517 (2007).

[8] Sankar D. S., Hemalatha K., “A non-Newtonian fluid flow model for blood flow through a catheterized artery—Steady flow”, Applied Mathematical Modeling, 31, pp.1847–1864 (2007).

[9] Mekheimer K. S., El Kot M. A., “Magnetic field and Hall currents influences on Blood flow through a stenotic artery”, Applied Mathematical Mechanics, English-Edition, 29, pp. 1093–1104 (2008).

[10] Mustapha N., Chakravarty S., Mandal P. K., Amin N., “Unsteady response of blood flow through a couple of irregular arterial constrictions to body acceleration”, Journal of Mechanics in Medicine and Biology, 8, pp. 395–420 (2008).

[11] Mustapha N., Amin N., Chakravarty S., Mandal P. K., “Unsteady magnetohydrodynamic blood flow through irregular multi-stenosed arteries”, Computers in Biology Medicine, 39, pp. 896–906 (2009). doi: 10.1016/j.compbiomed.2009.07.004 19665698 10.1016/j.compbiomed.2009.07.004

[12] Mustapha N., Amin N., Mandal P. K., Abdullah I., Hayat T., “Numerical simulation of generalized Newtonian blood flow past a multiple of irregular arterial stenosis”, Numerical Methods for Partial Differential Equations, 27, pp. 960–981 (2009).

[13] Abdullah I., Amin N., Hayat T., “Magnetohydrodynamic effects on blood flow through an irregular stenosis”, International Journal for Numerical Methods in Fluids, 67, pp. 1624–1636 (2011).

[14] Shahed S. R., Ali M., Saha S., Akhter M. N., “Numerical Study on unsteady flow field of arterial stenosis”, Procedia Engineering., 90, pp. 339–345 (2014).

[15] Sarkar A., Jayaraman G., “Correction to flow rate–pressure drop relation in coronary angioplasty: steady streaming effect”, Journal of Biomechanics, 31, pp. 781(1998). 9802778 10.1016/s0021-9290(98)00053-0

[16] Dash R. K., Jayaraman G., Mehta K. N., “Flow in a catheterized curved artery with stenosis”, Journal of Biomechanics, 32, pp. 49–61 (1999). 10050951 10.1016/s0021-9290(98)00142-0

[17] Reddy J. V. R., Srikanth D., Murthy S. V. S. S. N. V. G. K., “Mathematical modeling of pulsatile flow of blood through catheterized un-symmetric stenosed artery—Effects of tapering angle and slip velocity”, Eur. J. of Mech. B/Fluids, 48 (2014) 236–244.

[18] Srivastava V. P., Rastogi R., “Blood flow through a stenosed catheterized artery: Effects of hematocrit and stenosis shape”, Computers & Mathematics with Applications, 59, pp.1377–1385 (2010).

[19] Muthu P., kumar B. V. R., and Chandra P., “A study of Micropolar fluid in an annular tube with application to blood flow”, Journal of Mechanics in Medicine and Biology, 4, pp. 561–576 (2008).

[20] Thurston G. B., Henderson N. M., “The kinetics of viscoelastic changes due to blood clot formation”, Theoretical and Applied Rheology, 112, pp. 17–21 (1992).

[21] Chien S., King R. G., Skalak R., Usami S., Copley A. L., “Viscoelastic properties of human blood and red cell suspension”, Biorheology, 12, pp. 341–346 (1975). 1212514 10.3233/bir-1975-12603

[22] Thurston G. B., “Viscoelasticity of human blood”, Biophysics Journal, 12, pp. 1205 (1972). 10.1016/S0006-3495(72)86156-3 PMC1484135 5056964

[23] Thurston G. B., “Rheological parameters for the viscosity, viscoelasticity and Thixotropy of blood”, Biorheology, 16, pp. 149 (1979). 508925 10.3233/bir-1979-16303

[24] Yeleswarapu K. K., Kamaneva M. V., Rajagopal K. R., Antaki J. F., “The flow of blood in tubes: Theory and experiment”, Mechanics Research Communications, 25, pp. 257–262 (1998).

[25] Yeleswarapu, K. K., “Evaluation of continuum models for characterizing the constitutive behavior of blood”, Ph.D. thesis, University of Pittsburgh, (1996).

[26] Yilmaz F., Gundogdu M. Y., “A critical review on blood flow in large arteries; relevance to blood rheology, viscosity models, and physiologic conditions”, Korea-Australia Rheology Journal, 20, pp. 197–211 (2008).

[27] Canic S., Tambaca J., Guidoboni G., Mikelic A., Hartley C. J., and Rosenstrauch D., Modeling viscoelastic behavior of arterial walls and their interaction with pulsatile blood flow, Society for Industrial and Applied Mathematics, 67, pp. 164–193 (2006).

[28] Burton A. C., “Physiology and biophysics of the circulation” Introductory Text, Year Book Chicago: Medical Publisher, (1966).

[29] Massoudi M., Phuoc T. X., “Pulsatile Flow of a blood using second grade fluid model”, Computers and Mathematics with Applications, 56, pp. 199–211 (2007).

[30] Mandal P. K., “An unsteady analysis of non-Newtonian blood flow through tapered arteries with a stenosis”, International Journal of Non-Linear Mechanics, 40, pp. 151–164 (2005).

[31] Ikbal M. A., Chakravarty S., Mandal P. K., “Two-layered micropolar fluid flow through stenosed artery: Effect of peripheral layer thickness”, Computers and Mathematics with Applications, 230, pp. 243–259 (2009).

[32] K. A. Hoffmann, S. T. Chiang, Computational Fluid Dynamics, A Publication of Engineering Edition System, Wichita, Kansas USA, 1, pp. 67208–1078 (2000).

[33] Majhi S. N., Nair V. R., Pulsatile flow of third grade fluids under body acceleration modeling blood flow, Int. J. Eng. Sci. 32, pp. 839–846 (1994).

[34] Bugliarello G., Sevilla J., Velocity distribution and other characteristics of steady and pulsatile blood flow in fine glass tubes, Biorheol. 7, pp. 85–107 (1970). 10.3233/bir-1970-7202 5484335

[35] Shukla J. B., Parihar R. S., Rao B. R. P., Effects of peripheral layer viscosity on blood flow through the artery with mild stenosis, Bull. Math. Biol. 42, pp. 797–805 (1980). 7459493 10.1007/BF02461059

[36] Akay G., Kaye A., Numerical solution of time dependent stratified two-phase flow of micropolar fluids and its application to flow of blood through fine capillaries, Int. J. Eng. Sci., 23, pp.265–276 (1985).

[37] Pralhad R. N., Schultz D. H., Two-layered blood flow through stenosed tubes for different diseases, Biorheol. 25, pp. 715–726 (1988). 10.3233/bir-1988-25501 3252923

[38] Sajid M., Zaman A., Ali N., and Siddiqui A. M., Pulsatile Flow of Blood in a Vessel Using an Oldroyd-B fluid, I. J. Non. Sci. Num. Simu., 16, pp. 197–206 (2015).

[39] Srivastava V. P., Saxena M., Two-layered model of Casson fluid flow through stenotic blood vessels: Application to the cardiovascular system, J. Biomech. 27, pp. 921–928 (1994). 8063842 10.1016/0021-9290(94)90264-x

[40] Chaturani P., Palanisamy V., Pulsatile flow of power-law fluid model for blood flow under periodic body acceleration, Bio-rheo. 27, pp. 747–758(1990). 10.3233/bir-1990-27510 2271765

[41] Chaturani P., Palanisamy V., Pulsatile flow of blood with periodic body acceleration, Int. J. Eng. Sci. 29 pp. 113–121 (1991).

[42] Fung Y. C., Biomechanics Circulation, 2nd ed., Springer-Verlag, New York, USA, pp. 571 (1997).

